# Using Community Health Workers and a Smartphone Application to Improve Diabetes Control in Rural Guatemala

**DOI:** 10.9745/GHSP-D-20-00076

**Published:** 2020-12-23

**Authors:** Sean Duffy, Derek Norton, Mark Kelly, Alejandro Chavez, Rafael Tun, Mariana Niño de Guzmán Ramírez, Guanhua Chen, Paul Wise, Jim Svenson

**Affiliations:** a University of Wisconsin School of Medicine and Public Health, Department of Family Medicine and Community Health, Madison, WI, USA.; b University of Wisconsin School of Medicine and Public Health, Department of Biostatistics and Medical Informatics, Madison, WI, USA.; c University of California-Los Angeles David Geffen School of Medicine, Internal Medicine Residency Program, Los Angeles, CA, USA.; d Stanford University School of Medicine, Stanford, CA, USA.; e Hospital Obras Sociales Monseñor Gregorio Schaffer, San Lucas Tolimán, Guatemala.; f University of Wisconsin School of Medicine and Public Health, Department of Emergency Medicine, Madison, WI, USA.

## Abstract

**Background:**

The global prevalence of diabetes has nearly doubled since 1980. Seventy-five percent of patients with diabetes live in low- and middle-income countries, such as Guatemala, where health care systems are often poorly equipped for chronic disease management. Community health workers (CHWs) and mobile health technology have increasingly been applied to the diabetes epidemic in these settings, although mostly in supportive rather than primary roles in diabetes management. We sought to improve diabetes care in rural Guatemala through the development of a CHW-led diabetes program and a smartphone application to provide CHWs with clinical decision support.

**Methods:**

We worked with our local partners to develop a program model and the smartphone application (using the CommCare platform) and to train CHWs. We recruited patients with type 2 diabetes living in rural communities. Program evaluation used a single-group, pre-post design. Primary outcomes were hemoglobin A1c and the percentage of patients meeting A1c goals compared with baseline. We also followed a variety of process metrics, including application reliability.

**Results:**

Eighty-nine patients enrolled during the study period. The hemoglobin A1c percentage decreased significantly at 3 months (-1.0; 95% CI=-1.7, -0.6), 6 months (-1.5; 95% CI=-2.2, -0.8), 9 months (-1.3; 95% CI=-2.0, -0.6), and 12 months (-1.0; 95% CI=-1.7, -0.4). The percentage of patients with A1c ≤ 8% increased significantly at 3 months (23.6% to 44.4%, *P*=.007), 6 months (22.0% to 44.0%, *P*=.015), and 9 months (23.9% to 45.7%, *P*=.03). CHWs and supervising physicians agreed with application medication recommendations >90% of the time.

**Conclusion:**

Our results suggest that CHWs can safely and effectively manage diabetes with the assistance of a smartphone application and remote physician supervision. This model should be evaluated versus other standards of care and could be adapted to other low-resource settings and chronic diseases.

## Abstract (precis)

A smartphone application providing algorithmic clinical decision support enabled community health workers to improve diabetes control for a group of patients in rural Guatemala. This approach enables task sharing with physicians and other advanced practitioners for chronic disease care, which is particularly important in low-resource settings.

## Teaser

**Key Implications:**

- Program managers should consider equipping community health workers with clinical decision support applications to enable task sharing for chronic disease management.
- Researchers should examine the efficacy of this approach for chronic diseases other than diabetes and compared to traditional models of care.

Resumen en español al final del artículo.

## INTRODUCTION

The global prevalence of diabetes has increased dramatically over the past several decades, nearly doubling since 1980, from 4.7% to 8.5% of adults.^1^ In 2015, an estimated 5 million deaths and US$673 billion in health expenditures were attributable to diabetes, accounting for 12.8% of global all-cause mortality and 12% of global health expenditures.^2^ Low- and middle-income countries (LMICs), where 75% of people with diabetes now live and 80% of deaths due to diabetes occur, have been especially hard hit by this global epidemic. In addition to limited or episodic care, resources are scarce; in one study, only 29.6% of patients in low-income countries were currently taking diabetes medications compared with 74% in high-income countries.^3^

The World Health Organization (WHO) has advocated for the use of nonphysician health workers in the care of diabetes and other chronic diseases as a means to strengthen primary health care systems in LMICs.^1^ ^,^ ^4^ ^–^ ^8^ This approach is often referred to as task shifting, although there is a growing consensus that task sharing is a more appropriate framework given the difficulty of completely shifting highly complex clinical tasks to less extensively trained health care workers.^9^ ^,^ ^10^ Evidence is increasingly showing that sharing responsibilities with nonphysicians can improve access to care and patient outcomes for noncommunicable diseases.^9^ ^,^ ^11^ ^–^ ^15^ Community health workers (CHWs, also called health promoters)—lay people who are trained to carry out a variety of tasks and are often from or have a close connection to the communities they serve—are a common type of nonphysician health worker and are being increasingly utilized in health systems around the world, particularly in LMICs.^16^ ^,^ ^17^ Programs using CHWs for targeting diabetes care have shown improvements in glycemic control and other diabetes outcomes compared with standard care.^18^ ^–^ ^22^ In most of these programs, CHWs have played supportive roles (e.g., providing patient education or care coordination) rather than direct care roles.

With adequate training and support, CHWs have the potential to improve access to care and health care delivery. We evaluated the efficacy of trained CHWs enabled by a clinical decision support tool in directly providing elements of diabetes care with remote physician supervision, a novel approach for which little evidence exists at this time.

## METHODS

### Setting

This project occurred in the municipality of San Lucas Tolimán in the Western Highlands region of Guatemala, a middle-income country in Central America.^23^ A large majority of the population belongs to the Kaqchikel Mayan indigenous group. Poverty rates in this mostly rural municipality are high, with 91.1% of people living in poverty (<US$3 per day) and 29.4% in extreme poverty (<US$1.60 per day).^24^ A recent cross-sectional study conducted in San Lucas and other neighboring municipalities found a high prevalence of type 2 diabetes (13.8%) and prediabetes (13.8%).^25^

Our local partner was the San Lucas Mission (SLM), a nonprofit organization associated with the Catholic parish in San Lucas Tolimán providing health care and other social services to the estimated 34,713 people living in the municipality.^26^ A community needs assessment conducted in the summer of 2016 as part of the planning process for this project found that diabetes care in the rural villages was generally fragmented or inaccessible, medications and supplies were often in short supply, and patients had very limited diabetes-related knowledge, particularly with regard to self-care. Government-run rural health outposts ostensibly provide basic care for diabetes and other chronic diseases, but in reality, they are inadequately staffed and supplied. In San Lucas, they were not a reliable source of care for patients. While patients could seek care in the private sector, it was often cost prohibitive, particularly for medications. These challenges contributed to ineffective treatment: Only 58% of patients reported taking diabetes medications regularly, and only 13% were meeting blood glucose (BG) targets. These findings reflect prior analyses reporting poor access to effective diabetes care in Guatemala, particularly for rural, indigenous populations.^27^ ^,^ ^28^

Prior analyses found poor access to effective diabetes care in Guatemala, particularly for rural, indigenous populations.

SLM partners with local CHWs, known as promotores de salud (health promoters). These CHWs are recruited from the communities they serve, are bilingual in Spanish and Kaqchikel (the predominant Mayan language in this area), and generally have the equivalent of a US sixth grade education, affording basic literacy. General training for the CHW program occurs one weekend per month for 3 years and focuses on health prevention and early identification of patients for referral to a physician. The small group of leaders for the CHW program, called coordinators, are salaried. The other CHWs work mainly on a volunteer basis, but they receive a stipend per half day of work on dedicated health programs.

SLM had previously established an innovative and successful CHW-led childhood nutrition program enabled by mobile health technology.^29^ We sought to build on this foundation to create a sustainable rural diabetes program.

### Program Development

**FIGURE 1.**
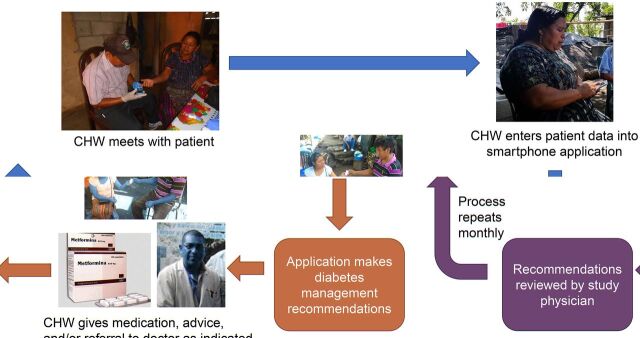
Overall Model for Sustainable Rural Diabetes Care Program Led by Community Health Workers, Guatemala

Program development was an iterative process that involved our local partners at all stages. We first developed an overall model for the program, as outlined in Figure 1. In this model, health promoters meet with patients on a monthly basis. The promoters use a clinical decision support (CDS) application to guide each visit. Using data entered by the promoters, including point-of-care glycemic testing, the application provides recommendations on the titration of oral hypoglycemics, management of diabetes complications, self-care counseling, and referral to the supervising physician. After each visit, patient data are uploaded to a secure server and reviewed by one of the supervising physicians, who then communicates any changes in the treatment plan or additional recommendations to the promoters. In order to remove cost as a barrier to care, the diabetes program provides services and medications free of charge.

We recognized that the services provided by this program, while intended to be an improvement on the status quo, were by no means comprehensive. Guidelines for limited resource settings also deem insulin, antihypertensives, and other therapies as essential elements of diabetes care.^30^ However, resources were not available to implement a comprehensive chronic disease system. Rather, glycemic control through oral medications and lifestyle counseling was deemed the immediate focus, with additional components to follow with enhanced resources and a successful mobile platform proof-of-concept.

### Development of Clinical Protocols and Procedures

We developed protocols for assessing glycemic control, titration of oral hypoglycemics, identification and management of diabetes complications, and patient counseling. We based these protocols on guidelines published by the American Diabetes Association (ADA),^31^ WHO,^32^ the International Diabetes Federation (IDF),^30^ and Guatemalan organizations.^33^ SLM medical director Dr. Rafael Tun was integral to this process and provided final approval for all protocols.

The mobile application included protocols for assessing glycemic control, titration of oral hypoglycemics, identification and management of diabetes complications, and patient counseling.

#### Assessment of Glycemic Control

We used point-of-care hemoglobin A1c (A1c) results as our primary measure of glycemic control based on recommendations from ADA and IDF.^30^ ^,^ ^31^ Studies have demonstrated the potential of this technology to improve diabetes care in LMICs.^33^ ^,^ ^34^ We utilized A1CNow+ (PTS Diagnostics) point-of-care capillary blood analyzers. The A1CNow+ test produces results in 5 minutes and can be performed with minimal training, allowing for assessment of glycemic control by the CHWs during diabetes visits. Guidelines recommend checking A1c every 2–6 months depending on diabetes control and changes in medication.^30^ ^,^ ^31^ We checked A1c every 3 months for all patients during the study period to allow for more uniform evaluation of program efficacy.

We also employed monthly BG testing to titrate medications between A1c measurements, assess for hypo- and hyperglycemia, provide a secondary marker of glycemic control when A1c testing was not available or malfunctioned, and as a confirmation of A1c values when checked concurrently. We used the Contour (Bayer) capillary blood testing system for BG testing. We established glycemic targets of A1c ≤7%, fasting BG 80–130 mg/dL, and postprandial BG <180 mg/dL for most patients, with less stringent targets for patients ≥65 years old or those with multiple comorbidities, or per physician discretion. These targets are broadly consistent with ADA and IDF guidelines.^30^ ^,^ ^31^

#### Medication Titration

We selected metformin and glyburide (glibenclamide) as the oral medications in our medication titration protocol because of their long track records in diabetes care, availability in Guatemala, and affordability. Metformin is the first-line medication for all patients, consistent with established guidelines,^30^ ^,^ ^31^ ^,^ ^35^ with glyburide added as a second agent when glycemic targets are not met. For patients with an initial A1c of ≥9%, the algorithm calls for dual therapy (metformin and glyburide), as recommended by ADA and American Association of Clinical Endocrinologists/American College of Endocrinology guidelines.^31^ ^,^ ^35^ The titration algorithm accounts for 4 factors in making medication recommendations: glycemic control, current medication dose(s), adherence, and side effects.

#### Identification and Management of Diabetes Complications

We developed protocols for common and important diabetes complications and comorbidities, including hyper- and hypoglycemia, hypertension, coronary artery disease, chronic kidney disease, diabetic foot ulcers, and diabetic eye disease (Table 1). These protocols include recommendations for referral to the supervising physician and, in some cases (e.g., hypoglycemia), initial treatment delivered by CHWs.

**TABLE 1.** Referral Protocols for Diabetes Complications and Comorbidities for Smartphone Application for Diabetes Care Program, Guatemala

**Routine Referrals (Within 1–2 Weeks)**	**Urgent Referrals (Within 1–2 Days)**	**Emergency Referrals (Same Day)**
Stage I hypertension (BP 140-160/90–100 mm Hg) Noninfected diabetic ulcer Need for renal function testing A1c ≥ 9% despite maximal doses of metformin and glyburide for ≥3 months A1c ≥ 9% for 3 consecutive checks A1c above glycemic target, but <9% for 4 consecutive checks Recent chest pain, moderate risk of CAD Blood in stool or possible melena Patient has other symptoms not addressed by the program protocols	Stage II hypertension (BP 160–200/100–120 mm Hg) Possibly infected diabetic ulcer, no signs of systemic infection Worsening vision FBG detectable, but ≥400 mg/dL A1c ≥ 14% Patient cannot tolerate minimum doses of metformin and/or glyburide Current chest pain, moderate risk of CAD	Severe hypertension (BP ≥ 200/120 mm Hg) Fasting blood glucose undetectable high Postprandial/random blood glucose undetectable high with mental status changes Hypoglycemia associated with altered mental status Persistent hypoglycemia despite treatment in the field Current chest pain, high risk of CAD Possibly infected diabetic ulcer with signs of systemic infection

Abbreviations: A1c, hemoglobin A1c; BP, blood pressure; CAD, coronary artery disease; FBG, fasting blood glucose.

### Application Development and Description

We integrated the diabetes protocols into a smartphone application to provide algorithmic decision support to the CHWs. The application also served as a data collection tool and medical record. We designed the application in Spanish for smartphones and tablets running the Android operating system (Google LLC), the most common mobile operating system in Guatemala^36^ and globally.^37^ We used devices with quad core processors and 1 GB of RAM. While most patient visits were conducted at least partly in Kaqchikel, we did not translate the application to Kaqchikel based on feedback from the bilingual CHWs because Kaqchikel is primarily a spoken language and most CHWs are literate only in Spanish.

In addition to providing algorithmic decision support to the CHWs, the application also served as a data collection tool and medical record.

Earlier versions of the application used Enketo (Enketo LLC) web forms for the user interface and Ona (Ona Systems) for data storage and management, which was then transitioned to the CommCare platform (Dimagi, Inc.), the most widely used mobile platform among frontline health workers in LMICs.^38^ While both platforms allow for offline data collection and have branching logic capabilities, permitting the delivery of algorithmic clinical decision support, we transitioned to CommCare because it has more advanced capabilities for storing and modifying longitudinal data, includes robust database functions, and allows for application updates to be pushed to end-user devices. To maintain data security, we encrypted and password protected all the smartphones running the application. CommCare is also password protected and uses AES 256-Bit Symmetric Encryption, a HIPAA-compliant encryption standard.

Prior to deployment in the field, we tested application language, workflow, and user interface with the CHWs and reviewed the embedded clinical algorithms to ensure that the application provided appropriate recommendations. We continued to elicit feedback from the CHWs and update the application throughout the study.

### CHW Training

**Figure:**
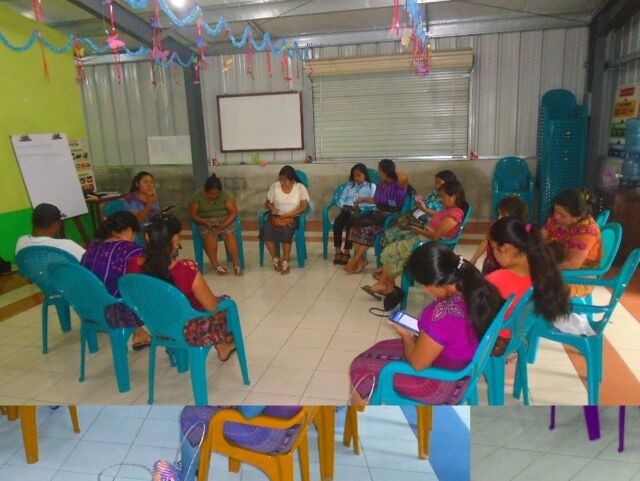
Community health workers in Guatemala practice using a smartphone application for diabetes care.Credit: © 2018 José Vicente Macario/San Lucas Mission

**Figure:**
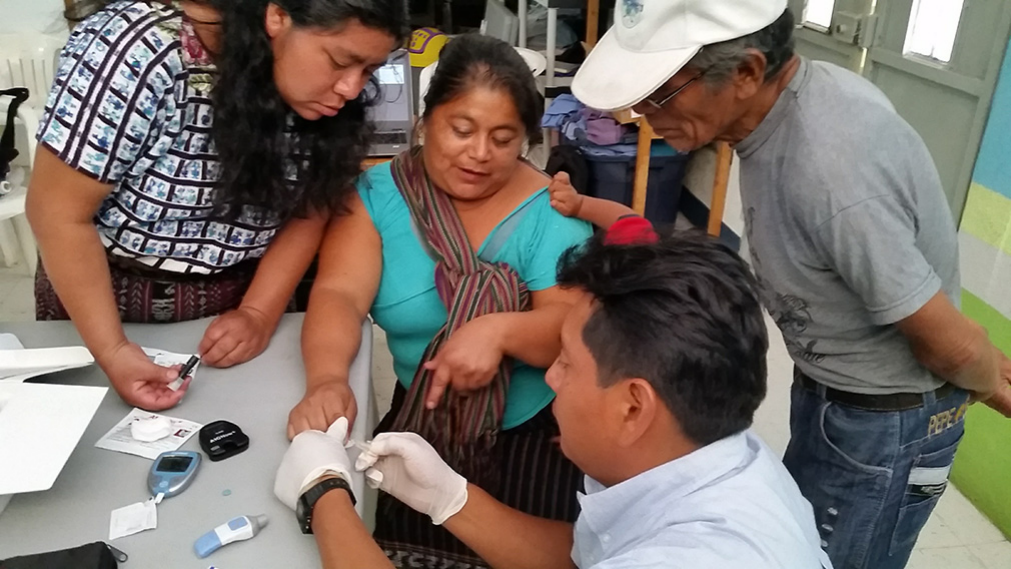
Community health workers in Guatemala practice point-of-care hemoglobin A1c testing.Credit: © 2017 Sean Duffy/University of Wisconsin

CHWs were recruited for participation from the general rural health promoter program. We trained these CHWs in basic diabetes care (including medication management, diabetes self-care and lifestyle counseling, and the recognition and management of complications), protection of human subjects, and use of testing equipment (e.g., glucometers) and the application. We adapted training materials regarding diabetes self-care developed by 2 other Guatemalan organizations that work with indigenous populations, Wuqu’ Kawoq^39^ and Hospitalito Atitlán.^40^ To learn how to conduct finger-stick testing using glucometers and the A1cNow+ device, measure blood pressure using automatic cuffs, and accurately measure height, weight, and waist circumference, CHWs first viewed a demonstration of these skills and then practiced in small groups. Application training consisted of one-on-one practice with a facilitator to simulate a patient visit.

CHWs were trained in basic diabetes care, protection of human subjects, and use of testing equipment and the application.

Total length of training was approximately 15 hours spread over several sessions. Dr. Duffy conducted the training sessions for the first several groups of CHWs. The coordinators of the CHW program led subsequent sessions. After receiving this initial structured training, CHWs were paired with one of the coordinators for patient care to continue supervised practice until they were able to complete a visit with minimal direction, a process which generally took 15 patient visits (approximately 9 hours).

### Program Evaluation

#### Study Design

We used a single group, pre- and posttest design. Inclusion criteria for the program were established type 2 diabetes and age greater than 18 years. Exclusion criteria were insulin therapy, pregnancy, renal insufficiency (defined as estimated glomerular filtration rate [GFR] <30 mL/min/1.73 m^2^), and physician discretion.

#### Clinical Outcome Measures

Primary clinical outcomes were A1c and the percentage of patients with A1c ≤8% and meeting individual A1c goals compared with baseline. When A1c was higher than the detectable range of the A1CNow+ analyzer (displayed as “>13.0%”), we imputed these values conservatively as 13.1%. Secondary outcomes included BG, blood pressure, weight, body mass index (BMI), and waist circumference. When BG was higher than the detectable range of the Contour glucometers (>600 mg/dL, displayed as “HI”), we also imputed these values conservatively as 601 mg/dL.

We tracked the prevalence of medication side effects, change in medication dose, complications of diabetes and related referrals, and adverse events, with a focus on hypoglycemia (defined as BG <70 mg/dL) and hypoglycemia symptoms.

#### Behavioral Outcome Measures

We administered validated Spanish versions of 2 standardized questionnaires—the Diabetes Knowledge Questionnaire (DKQ)^41^ and the Summary of Diabetes Self-Care Activities (SDSCA)^42^ ^,^ ^43^—in June 2018 to 2 subgroups of patients: patients enrolled in the past 3 months and those who had been participating for 6 months or more. We repeated questionnaires for patients in the newly enrolled group in January 2019.

#### Application-Specific and Process Outcomes

For each visit, we tracked whether the CHWs and the supervising physician reviewing visit data agreed with medication recommendations provided by the application. We also tracked instances in which the application provided erroneous recommendations (as determined by the physician reviewing visit data). We administered a Spanish translation of the System Usability Scale, the most widely used standardized usability questionnaire, to all CHWs who had used the application. This scale results in a usability score from 0 to 100. We used a grading schema proposed by Bangor et al.,^44^ which rates usability scores less than 50 as “not acceptable,” those between 50 and 70 as “marginally acceptable,” and scores above 70 as “acceptable.” This usability survey also solicited written feedback about the application. Finally, we maintained detailed records of program costs in order to estimate the average cost per patient.

For each visit, we tracked whether the CHWs and the supervising physician agreed with medication recommendations provided by the application.

#### Patient Recruitment

Based on cases known to the CHWs, we estimated the number of patients with diagnosed diabetes in the rural communities of interest to be approximately 150. The CHWs recruited these patients for the program and we set an enrollment target of 100 patients, which reflected the resources and CHW capacity available for the program.

#### Statistical Analysis

We used R (The R Foundation) for analysis of program outcomes. We analyzed differences in continuous variables (e.g., A1c) using generalized additive mixed effects models (GAMMs) with the nonlinear smoothing function on time since program enrollment. For all health outcomes, baseline covariates of age, sex, and years since the participant’s diabetes diagnosis were included as standard main effects; the penalized regression splines were used on the longitudinal covariate of time since enrollment. Models also included subject-specific random intercepts and time-since-enrollment slopes. For the glucose outcome, whether the participant had been fasting at the time of measurement was also included in the models as a longitudinal main effect.

For the outcomes of A1c and glucose, values that were at the limit of detection were treated as a typical value in these GAMMs. In order to test if significant change in health outcomes from baseline occurred at 3, 6, 9, and 12 months after enrollment, bootstrapped confidence intervals were employed. Due to the censored nature of some of the A1c and glucose values, a sensitivity analysis of these outcomes was conducted using a Cox proportional hazard mixed effects modeling structure. Model diagnostics revealed a concern for heteroscedasticity in the glucose model. Refitting the model on the natural-log of glucose alleviated the issue, thus all reported glucose modeling results are from a model fitted to the natural-log of glucose.

The same GAMM structure already described was used to analyze A1c control (≤8%) and A1c goal attainment separately, with the appropriate model setup changes for the outcome being binary instead of continuous. Additionally, for A1c control/goal attainment, a pre-post study design was mimicked within the data by selecting each participant’s baseline value and their value closest to the 3, 6, 9, and 12 month follow-up period (within ±45 days, otherwise the observation at follow-up was considered missing). These pairs were then used to perform a McNemar test on the change in A1c control/goal attainment at these 4 follow-up times.

For DKQ and SDCA scores and medication doses, we used the Shapiro-Wilk test to determine normality. We then used 2-tailed *t* tests to assess differences in normally distributed variables and the Wilcoxon test for nonnormally distributed variables. We used a significance threshold (α) of 0.05 for all analyses.

### Ethical Oversight and Funding

The program was reviewed and approved by the University of Wisconsin and Stanford University institutional review boards, as well as the SLM Health Program. All patients provided written informed consent after a bilingual CHW explained the study and risks and benefits of participation in the patients’ preferred language (Spanish or Kaqchikel). A seed grant from the University of Wisconsin Global Health Institute provided funding.

## RESULTS

### Enrollment and Retention

Eighty-nine patients enrolled during the study period (February 2017 to June 2019), and 67 remained in the program at the end of this period (retention rate of 75.3%). Of patients who completed at least one follow-up visit, median follow-up time was 12.1 months (range 1.1–28.2, IQR 9.8). One patient died while participating in the program, 2 withdrew, and 11 were lost to follow-up. Eight patients were excluded after enrollment, 4 because of renal failure, 1 because of recurrent hypoglycemia while taking metformin alone, 1 because of hyperglycemia requiring insulin therapy, and 2 because of terminal illness.

Patients completed 920 visits (enrollment and monthly), 80.8% occurring at the designated central location and 19.2% in patient homes. Patients who remained in active follow-up completed 93.8% of possible visits, with all patients (including those who were excluded or lost to follow-up) completing 80.7% of possible visits.

**Figure:**
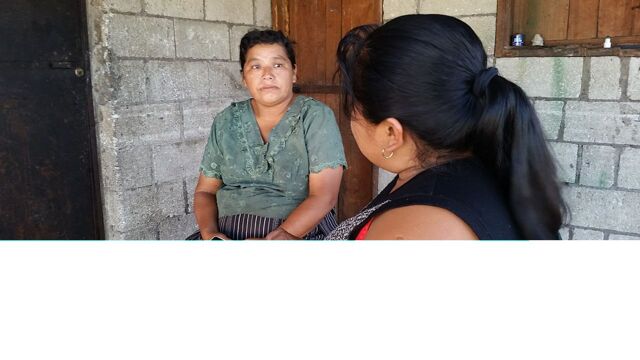
A community health worker in Guatemala conducts a home visit with a diabetes patient using a smartphone application for clinical decision support.Credit: © 2017 Sean Duffy/University of Wisconsin

### Cohort Profile

Table 2 summarizes the baseline characteristics of enrolled patients, including place of diagnosis (a proxy for prior source of care) and medication use. Of note, a large majority (82%) of enrolled patients were women. Baseline glycemic control was poor, with a mean A1c (standard deviation [SD]) of 10.0% (2.5) and only 20% of patients meeting A1c treatment goals.

**TABLE 2.** Baseline Characteristics of Patients Enrolled in a Rural Diabetes Care Program, Guatemala

**Characteristic (N=89)**	**Value**
Demographics	
Mean age (SD), years	53.5 (13.3)
Sex, % female	82
Years since diabetes diagnosis, median (IQR)	4 (6)
Place of diagnosis, %	
San Lucas Mission rural clinic	40
Private clinic	20
Nongovernmental organization hospital	16
Guatemalan Social Security clinic	12
Government clinic	6
Other	6
Medication use, %	
Taking any diabetes medication^a^	82
Metformin	71
Glyburide	30
Glimepiride	3
Natural remedies	18
Clinical measures	
Mean hemoglobin A1c (SD), %	10.0 (2.5)
Proportion with A1c at goal, %	20
Mean body mass index (SD), kg/m2	26.7 (4.6)
Mean blood glucose (SD), mg/dL	237 (126)

Abbreviations: IQR, interquartile range; SD, standard deviation.

a Does not include natural remedies.

Baseline glycemic control was poor, with a mean A1c of 10.0% and only 20% of patients meeting A1c treatment goals.

### Clinical Outcomes

GAMM regression results are displayed in the Supplement. Age at baseline was significantly associated with A1c (β=−0.046, *P*=0.002), natural-log glucose (β=−0.008, *P*= 0.003), systolic blood pressure (β=0.569, *P*<0.001), A1c control (OR=1.05, *P*=0.005), and A1c goal attainment (OR=1.08, *P*<0.001) but not associated with diastolic blood pressure, weight, waist circumference, or BMI. Baseline years since diabetes diagnosis was significantly associated with A1c (β=0.073, *P*=0.021), natural-log glucose (β=0.018, *P<*0.001), A1c control (OR=0.90, *P*=0.005), and A1c goal attainment (OR=0.89, *P*=0.025), but no other health outcomes. Fasting status was only in the glucose model and was significantly associated with natural-log glucose (β=−0.357, *P*<0.001). Sex was not associated with any of the health outcomes examined.

**FIGURE 2.**
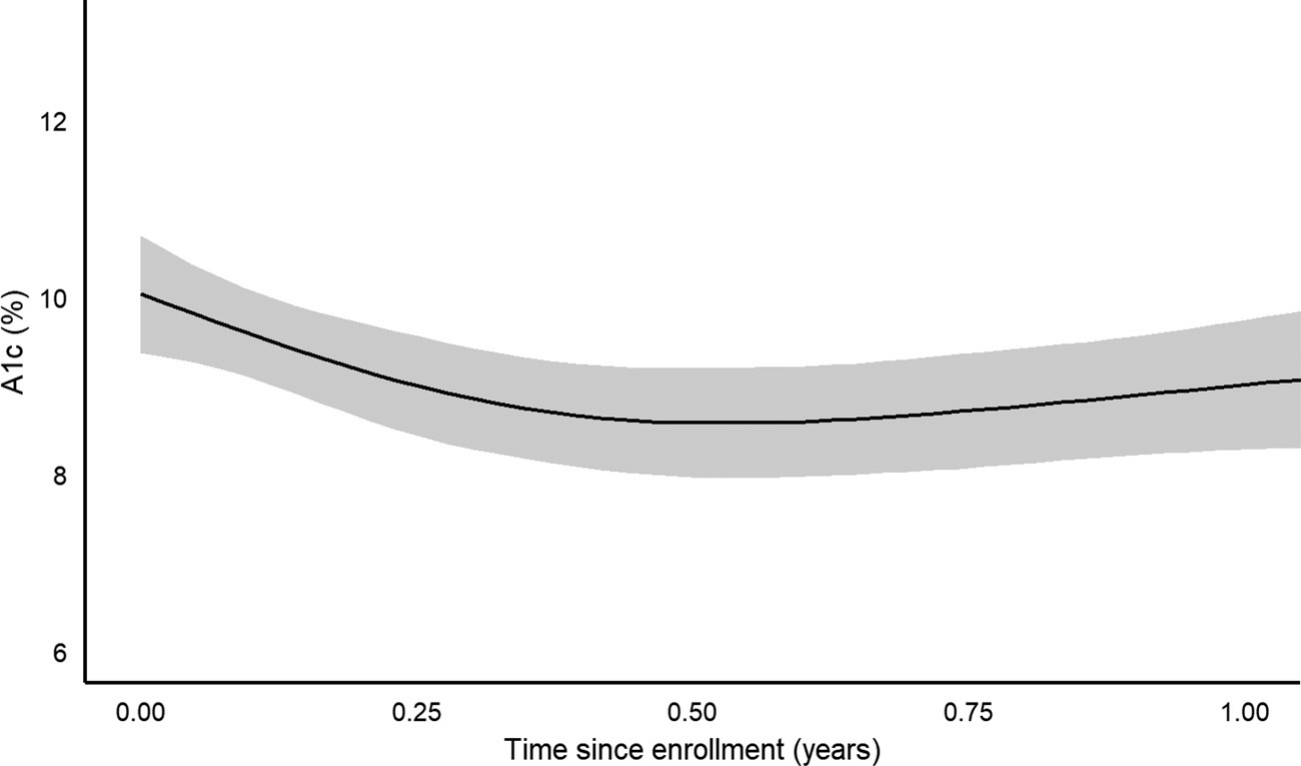
Estimated Change in Hemoglobin A1c Over Time After Enrollment in Rural Diabetes Care Program, Guatemala

**FIGURE 3. fig3:**
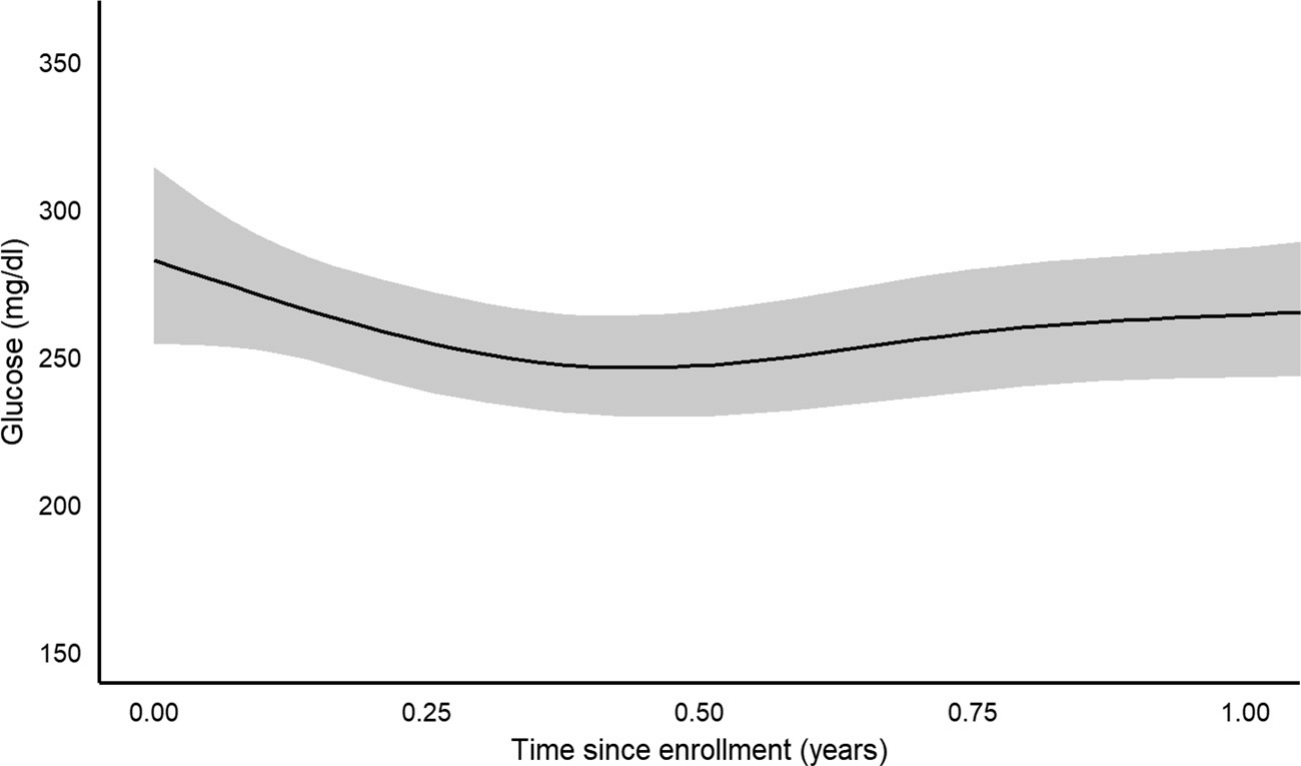
Estimated Change in Glucose Over Time After Enrollment in Rural Diabetes Care Program, Guatemala

**FIGURE 4.**
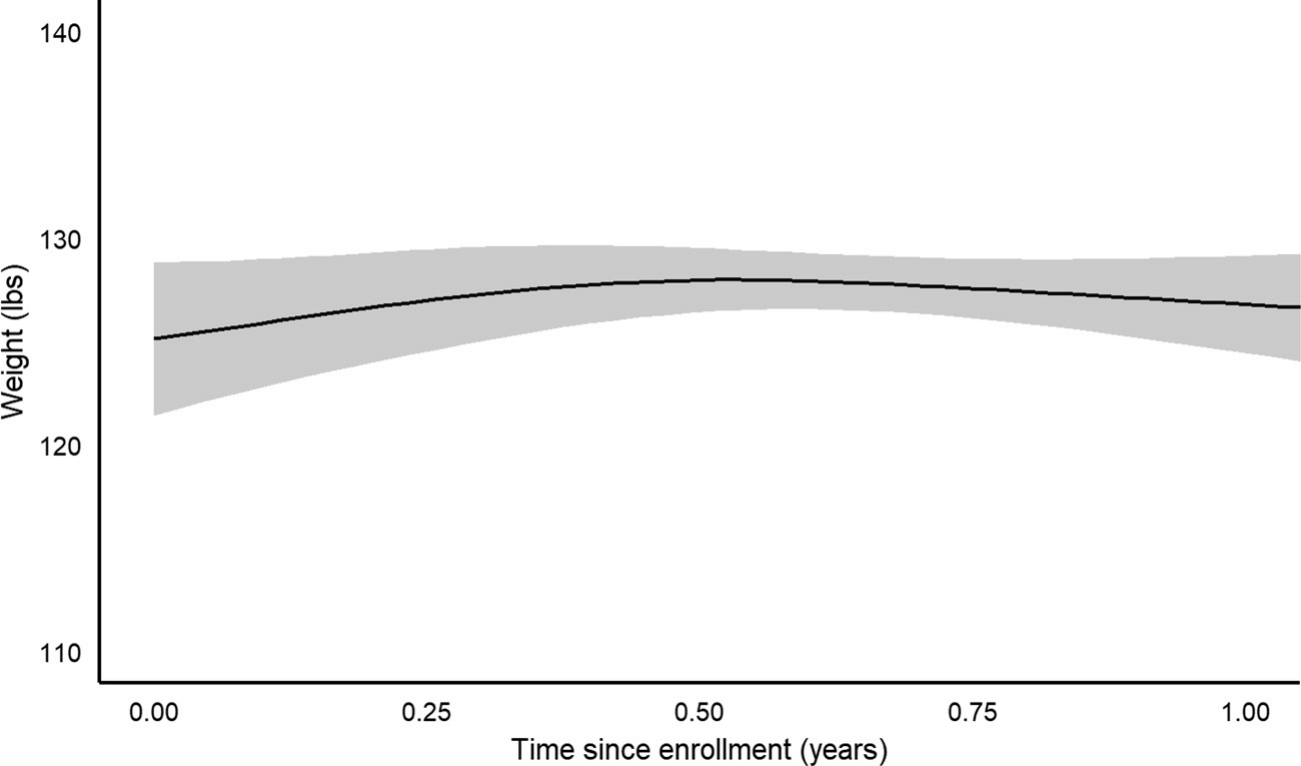
Estimated Change in Weight Over Time After Enrollment in Rural Diabetes Care Program, Guatemala

Time since program enrollment was significantly associated with the outcomes of A1c, natural-log glucose, weight, and BMI (all *P*<0.001), with nonlinear behavior between times since enrollment and these outcomes. Figures 2 [Fig fig3] to 4 show the estimated behavior over time for A1c, glucose, and weight; BMI and weight results were very similar to one another, as expected, and only the weight figure is shown. Both A1c and glucose were estimated to decrease up to around 6 months, and then slowly rise back towards baseline values afterwards. However, the sparsity of observations after 1 year resulted in increased uncertainty in the estimated trend after this point. Both weight and BMI were estimated to increase up to about 6 months after baseline, then to slowly decrease afterwards. After 1 year, the uncertainty in the estimation of the trend increased greatly.

Based on the bootstrapped results for changes from baseline at 3, 6, 9, and 12 months (Table 3), A1c displayed significant reductions from baseline at all 4 intervals, with the greatest estimated reduction at 6 months (1.45 A1c % mean reduction), but still a 1-point reduction estimated at 1 year after enrollment. Natural-log glucose displayed significant reductions from baseline at 3 and 6 months, with the greatest estimated reduction at 6 months (0.135 natural-log mL/dL mean reduction; 22.4 mL/dL reverse transformed for a typical subject in the data; see Table 3 footnote), but the reduction at 9 and 12 months was nonsignificant. Weight displayed a significant mean gain over baseline at 3 months (1.86 lb estimated gain), but no significant change from baseline at the other time points. BMI displayed a similar trend to weight.

**TABLE 3.** Bootstrapped Results for Change Since Baseline in Outcomes Among Patients Enrolled in Rural Diabetes Care Program, Guatemala

**Outcome**	Time Since Baseline	Estimated Change	95% CI Lower Bound	95% CI Upper Bound
A1C, %	3 months	–1.04^a^	–1.68	–0.559
	6 months	–1.45^a^	–2.19	–0.813
	9 months	–1.32^a^	–2.01	–0.636
	12 months	–1.03^a^	–1.73	–0.385
Glucose, natural-log mL/dL	3 months	–0.104^a^	–0.199	–0.0244
	6 months	–0.135^a^	–0.232	–0.0366
	9 months	–0.0909	–0.163	0.00368
	12 months	–0.0677	–0.166	0.0175
Glucose,^b^ mL/dL	3 months	–17.5^a^	–36.3	–5.00
	6 months	–22.4^a^	–39.2	–5.20
	9 months	–15.4	–27.3	1.70
	12 months	–11.6	–30.7	3.28
Systolic BP, mm Hg	3 months	0.375	–5.02	1.08
	6 months	0.75	–3.62	2.48
	9 months	1.13	–2.84	3.87
	12 months	1.5	–2.82	3.83
Diastolic BP, mm Hg	3 months	–0.0678	–0.812	1.1
	6 months	–0.189	–1.22	1.77
	9 months	–0.467	–1.83	1.74
	12 months	–0.877	–2.59	1.02
Weight, lb	3 months	1.86^a^	0.355	3.29
	6 months	2.84	–0.0432	5.17
	9 months	2.44	–1.67	4.88
	12 months	1.67	–3.89	4.77
Waist-circumference, cm	3 months	0.269	–0.474	1.28
	6 months	0.51	–0.677	1.86
	9 months	0.718	–0.632	1.99
	12 months	0.896	–0.253	2.19
BMI, kg/m^2^	3 months	0.372^a^	0.0856	0.681
	6 months	0.616^a^	0.0477	1.1
	9 months	0.639	–0.229	1.14
	12 months	0.538	–0.674	1.1
**Outcome**	**Time Since Baseline**	**Est. Difference in Probability of Control/Goal**	**95% CI Lower Bound**	**95% CI Upper Bound**
A1C control, A1C ≤ 8%	3 months	0.127	–0.0238	0.276
	6 months	0.203	–0.0472	0.454
	9 months	0.205	–0.0702	0.391
	12 months	0.166	–0.0927	0.341
A1C goal, A1C ≤ subject goal	3 months	0.0588	–0.0096	0.22
	6 months	0.0999	–0.0188	0.394
	9 months	0.106	–0.0276	0.263
	12 months	0.0929	–0.0359	0.195

Abbreviations: A1C, hemoglobin A1C; BMI, body mass index; BP, blood pressure; CI, confidence interval.

a *P*<.05.

b As these numbers are no longer on the scale of the regression, these values are specific to the type of subject the predictions were performed on (i.e., the values the other covariates are set at for prediction affect these numbers, unlike on the regression scale), which was the most common subject sex (female) and fasting value (true), median baseline age (54 years), and median years since diabetes diagnosis at baseline (4 years) for the subjects in analyses.

**FIGURE 5.**
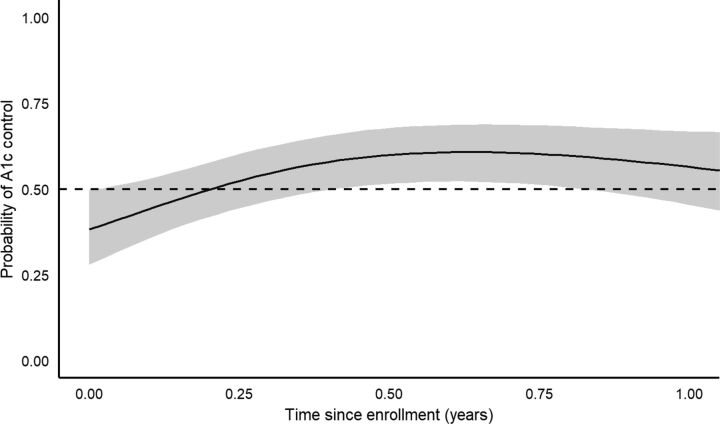
Estimated Probability of Hemoglobin A1c Control (≤8%) Over Time After Enrollment in Rural Diabetes Care Program, Guatemala

**FIGURE 6.**
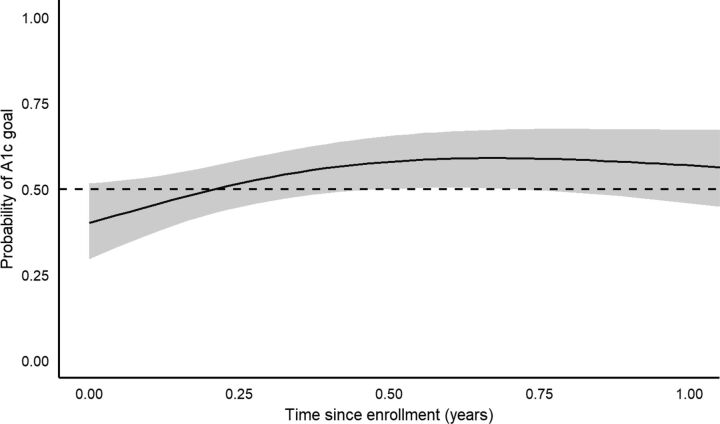
Estimated Probability of Meeting A1c Goal Over Time After Enrollment in Rural Diabetes Care Program, Guatemala

GAMM results for A1c control/goal attainment (Figures 5 and 6) showed an initial trend towards increased attainment until approximately 6 months, followed by a trend back towards baseline. No significant association was found between time since enrollment and probability of A1c control/goal attainment. However, when mimicking a pre/post design and analysis for examining these outcomes at 3, 6, 9, and 12 months from baseline, time periods closer to baseline were associated with significant increases in the proportion with A1c control/goal attainment (Table 4), similar to the A1c continuous analyses above. For A1c control, significant proportion increases from baseline were detected at 3, 6, and 9 months after baseline (*P*-values<0.034), but not at 12 months (*P*=0.121). For A1c goal attainment, significant proportion increases from baseline were detected at 3 and 6 months (*P*-values<0.020), but not at 9 and 12 months (*P*-values>0.114). However, for both outcomes and at all 4 follow-up periods, the raw proportion increased, ranging from a 17.1% to 22.0% increase in A1c control, and from 7.3% to 20.0% increase in A1c goal attainment.

**TABLE 4.** Change in Proportion of Patients Meeting A1c Targets Among Those Enrolled in Rural Diabetes Care Program, Guatemala

**Outcome**	**Time Since Baseline**	**N**	**Pre Control, %**	**Post Control, %**	**Proportion Change, %**	**McNemar *P* Value**
A1c control	3 months	72	23.6	44.4	20.8	.007
	6 months	50	22	44	22	.015
	9 months	46	23.9	45.7	21.8	.034
	12 months	41	26.8	43.9	17.1	.121
						
**Outcome**	**Time Since Baseline**	**N**	**Pre at Goal, %**	**Post at Goal, %**	**Proportion Change, %**	**McNemar *P* Value**
A1c at goal	3 months	72	16.7	31.9	15.2	.015
	6 months	50	14	34	20	.016
	9 months	46	15.2	28.3	13.1	.114
	12 months	41	17.1	24.4	7.3	.55

Abbreviation: A1c, hemoglobin A1c.

Sensitivity analyses using Cox mixed effects models to handle the true censored nature of the A1c and glucose values had numerous issues with the assumptions of proportionality. The results of the A1c model confirmed the GAMM A1c results, with increased time since enrollment associated with decreased A1c values (a significant “increased hazard” of observing A1c at lower values). The glucose model did not display a significant association between glucose and time since enrollment. However, from the GAMM results, there appeared to be nonlinear behavior between glucose and time. The Cox model did not properly account for this nonlinearity, and the “fall then rise” nature of the trend paired with the assumption violations could obscure a true association.

The GAMM results did not show any significant relationship between time since enrollment and blood pressure or waist circumference, and the bootstrapped results did not show any significant difference in these variables at the designated analysis time points. We also ran unadjusted analyses for all the models described above, which were consistent with the adjusted results in terms of statistically significant associations and the nonlinear forms between time since intervention and outcomes.

### Medication Titration, Side Effects, and Adverse Events

Median daily doses of metformin and glyburide increased significantly (all *P*≤0.02) from pre-enrollment to first recommendation at enrollment visit (500 to 1,700 mg and 0 to 2.5 mg) and from enrollment visit to last visit (1,700 to 2,550 mg and 2.5 to 5 mg). Patients taking metformin reported typical gastrointestinal side effects during 6.7% of visits. Side effects were significant enough to warrant a dosage reduction per the titration protocol during 3.9% of metformin-exposed visits (29.9% of metformin-exposed patients, 0.5 events per patient-year of therapy). There were 11 episodes of documented hypoglycemia (BG <70 mg/dL). Glyburide dosage was reduced due to hypoglycemia symptoms or documented hypoglycemia for 7.8% of glyburide-exposed visits (36.1% of glyburide-exposed patients, 0.9 events per patient-year of therapy).

Nine of the 11 hypoglycemic episodes were mild and resolved with treatment by CHWs or at home. Two hypoglycemic episodes required hospitalization for management. Both episodes occurred in the same patient, who was taking metformin alone and also had concomitant severe acute illnesses at the time of the episodes.

### Complications of Diabetes

Forty-four patients (49.1%) were identified as having increased risk of chronic kidney disease. Of these patients, 35 (80%) underwent renal function testing. Mean (SD) GFR was 77.1 (34.7) mL/min/1.73 m^2^. Twenty-six (74.3%) patients in this group had normal GFR (>60 mL/min/1.73 m^2^), 5 (14.3%) had GFR 30–60 mL/min/1.73 m^2^, and 4 (11.4%) had significantly reduced renal function with GFR <30 mL/min/1.73 m^2^.

**FIGURE 7.**
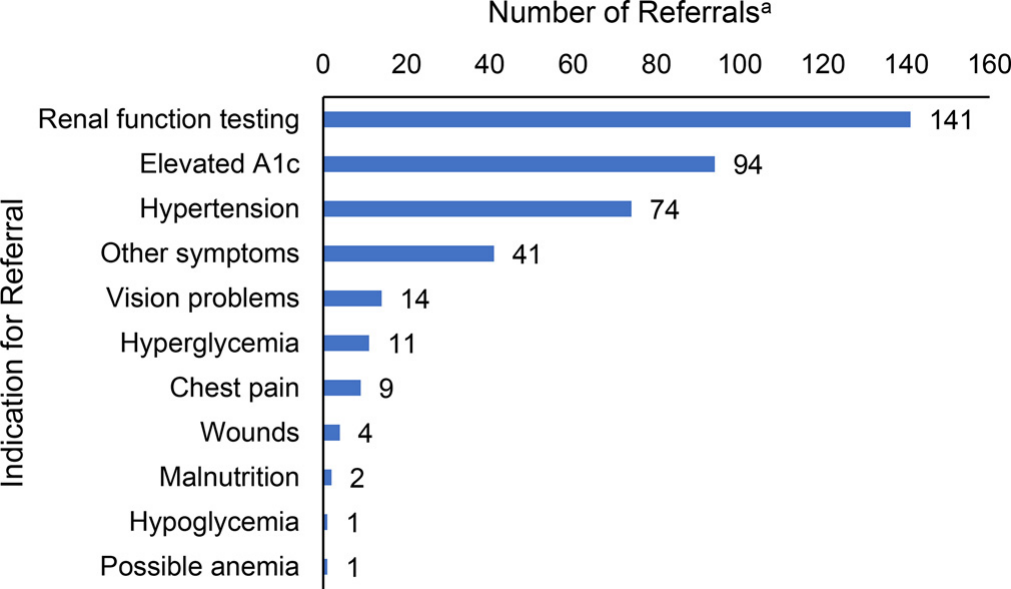
Referrals Recommended by Smartphone Application to Supervising Physician by Indication, Guatemala^a^ ^a^ Sum of indications is greater than total number of individual referrals (279) as some referrals had multiple indications.

A total of 279 referrals were recommended by the application for one or more potential complications of diabetes, representing 30.3% of visits. Of these, patients accepted 134 (48.0%) referrals. Based on available referrals tracking data, we estimate that patients completed 50.0% of accepted referrals, representing 24.0% of all recommended referrals. Figure 7 lists referrals by indication. Renal function testing was by far the most common indication for referral (50.5% of all referrals). Our clinical algorithms call for repeat referrals for renal function testing until completed for patients for whom it is indicated, contributing to the high number of referrals for this indication.

One patient died while participating in the program. The probable cause of death was myocardial infarction. This patient had well-controlled diabetes on metformin alone and had not reported symptoms of myocardial ischemia or other complications prior to their death.

### Behavioral Outcomes

Thirteen patients who had been in the program for 6 months or more and 11 patients who had enrolled in the past 3 months completed the DKQ and SDSCA. DKQ scores did not vary between the 2 groups, with a mean score of 13 for both (*P=*1). Of the newly enrolled patients, we were able to repeat the DKQ 6 months later for 5 patients. There was no significant difference between baseline and follow-up scores (mean 13 vs. 12.8, *P*=1).

Patients who had been enrolled for at least 6 months had higher average SDSCA scores (scored 0 to 7, with 7 being optimal) in several self-care categories compared with newly enrolled patients (see Table 5). However, only differences in foot care and dedicated exercise were statistically significant, with dedicated exercise scores being better in the newly enrolled group. We obtained follow-up SDSCA scores for 5 patients in the recently enrolled group 6 months after the initial questionnaire, which did not show any statistically significant improvements.

**TABLE 5.** Comparison of Summary of Diabetes Self-Care Activities (SDSCA) Between New and Established Patients^a^

**Measure**	Patients Enrolled ≥6 Months (n=13)	Patients Enrolled <3 Months (n=11)	***P* Value**
Healthy diet in the past week	7.0 [1.0]	6.0 [3.0]	.294
Healthy diet in general	7.0 [1.0]	6.0 [2.5]	.310
Eating fruits and vegetables	4.2 (2.1)	3.2 (2.5)	.319
Avoidance of high-fat foods	7.0 [1.0]	6.0 [0.5]	.088
Even distribution of carbohydrates	7.0 [0.0]	7.0 [0.0]	.755
Specific diet score	5.3 (1.1)	4.5 (1.1)	.088
General diet score	7.0 [1.0]	6.0 [2.8]	.336
Physical activity	7.0 [0.0]	7.0 [2.0]	.414
Dedicated exercise	0.0 [0.0]	0.0 [1.0]	.020
Exercise subscore	3.5 [0.0]	3.5 [1.5]	.424
Foot care	7.0 [1.0]	4.0 [5.5]	.047
Medication adherence	7.0 [0.0]	7.0 [0.0]	.849

a Values with parentheses represent mean (SD) and those with brackets represent median [IQR].

### Application-Specific and Process Outcomes

**Figure:**
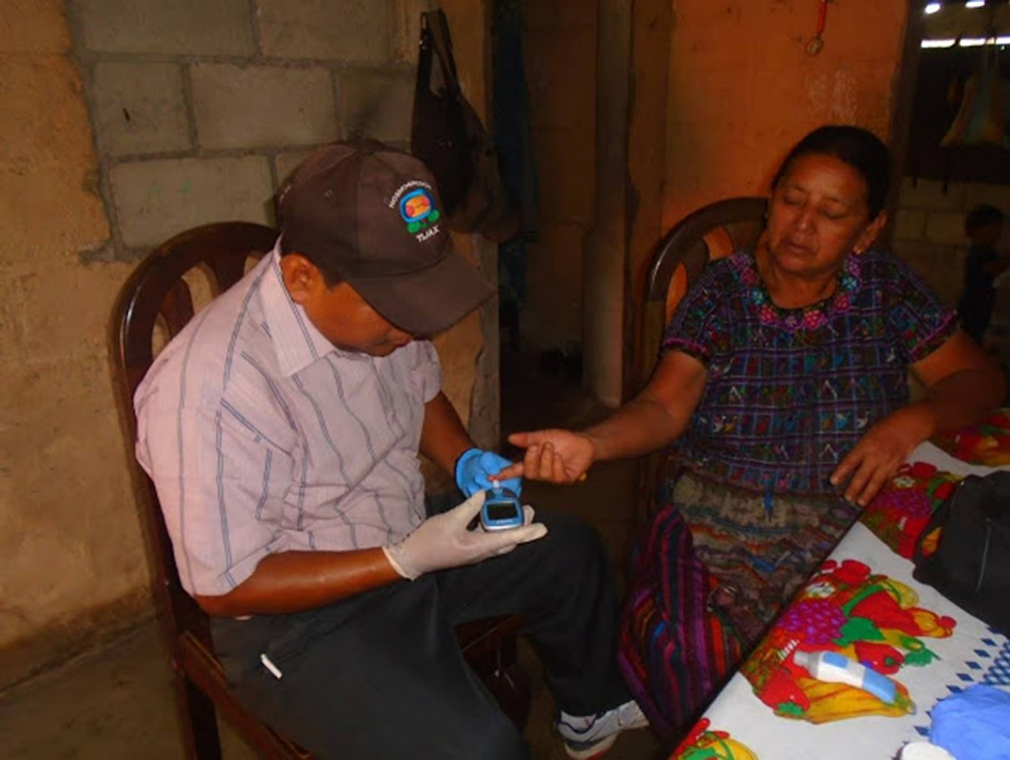
A community health worker in Guatemala checks the blood glucose of a diabetes patient during a home visit.Credit: © 2018 Cesia Castro Chutá/San Lucas Mission

CHWs and the reviewing physician agreed with medication recommendations given by the application for 90.9% of visits. During 53 visits (5.8%), medication recommendations were altered by the CHWs after remote consultation with a physician. The reviewing physician changed medication recommendations based on data review after 30 visits (3.3%). There were 4 cases in which the application made inappropriate recommendations or malfunctioned. In each of these cases, patient treatment was corrected through direct communication between the supervising physician and the CHWs and future errors were prevented through application updates.

CHWs and the reviewing physician agreed with medication recommendations given by the application for 90.9% of visits.

Twenty-one CHWs completed the System Usability Scale survey in January 2019. The mean score for fully completed surveys was 61.3 (range 27.5–87.5) and the mean composite score (accounting for responses from partially completed surveys) was 62.1. Subgroup analysis of scores above and below the predefined “acceptable” threshold of 70 showed that CHWs who rated application usability above 70 (n=4) were younger (mean age 32.0 vs. 42.2 years), more educated (mean 10.2 vs. 5.8 years of education), used smartphones more often (median use daily vs. once weekly), and had greater experience with the diabetes application (median use 11–15 times vs. less than 5 times) on average than those with scores 70 or below (n=12). Fourteen CHWs provided written subjective feedback on how the application could be improved. Common recommendations for improvement were to make the application faster and more responsive, reduce the number of questions and simplify language, and increase the amount of practice that CHWs had with the application.

We estimated a program start-up cost of US$3,940 for 100 patients, with continuing costs of US$118 per patient, per year (Table 6).

**TABLE 6.** Estimated Program Start-Up and Maintenance Costs

Start-Up Costs^a^	
*Expenditure*	*Total cost*
Glucometers and lancing devices	$140
Smartphones	$600
Automatic blood pressure cuffs	$200
CommCare fees^b^	$3,000
Total	$3,940
**Continuing per Capita Costs**	
*Expenditure*	*Cost per patient, per year*
Medications (metformin, glyburide, aspirin)	$32
Hemoglobin A1c tests	$38
Other testing supplies (e.g., glucose strips, lancets)	$9
CHW labor costs	$16
CHW coordinator labor costs	$13
Other costs (e.g., equipment replacement, data plan^c^)	$10
Total	$118

Abbreviation: CHW, community health worker.

a Reflects start-up costs for an anticipated patient population of 100 patients.

b This reflects current CommCare fees, which are $250/organization/month for a basic plan. CommCare fees are not reflected in continuing per capita costs because they are not dependent on caseload, and in our case, they support other health programs with thousands of total patients.

c CommCare projects generally use 100 MB or less of mobile data per month.

## DISCUSSION

Our results from the development and implementation of this program suggest that CHWs enabled by CDS technology can safely and effectively manage diabetes in rural Guatemala with remote physician supervision. Longitudinal analysis demonstrated significant improvements in the primary outcome of A1c, including at the predefined time points of 3, 6, 9, and 12 months after program enrollment. Statistically significant improvements in A1c ranged from 1.0% to 1.4%. These A1c improvements also meet the commonly used threshold of 0.5% for a clinically significant change in A1c.^45^ ^,^ ^46^

Our results suggest that CHWs enabled by CDS technology can safely and effectively manage diabetes in rural Guatemala with remote physician supervision.

The proportion of patients with A1c ≤8% and meeting individualized treatment goals increased at each of these time points as well, with statistically significant increases at 3, 6, and 9 months and 3 and 6 months, respectively. However, it should be noted that significant covariates of age and years since diabetes diagnosis were not accounted for in these results. The GAMM analyses, which included these covariates, showed a trend in A1c control/goal attainment similar to that in the continuous A1c analysis, but the control/goal attainment trend did not meet statistical significance. Given that the continuous GAMM models showed significant improvements in A1c over time, the failure to detect statistically significant improvements in the adjusted binary A1c outcomes could have been a function of inadequate power.

The improvements in glycemic control associated with this program are similar to those reported for other CHW-led diabetes interventions in LMICs.^21^ ^,^ ^47^ ^,^ ^48^ A key difference from prior published interventions using CHWs in diabetes care is that rather than providing ancillary services, such as patient education, in support of traditional medical care, CHWs in our program were directly providing care: they assessed glycemic control, directed medication therapy, and identified potential complications with the assistance of mobile CDS technology. This approach is relevant for similar LMIC settings around the world, where health systems are faced with a rising tide of diabetes and other chronic diseases in the context of dire shortages of physicians, nurses, and other highly trained health workers.^7^ ^,^ ^49^

### Decision Support

In general, the application provided reliable recommendations, with CHWs and the reviewing physician agreeing with the application-recommended medication dosing greater than 90% of the time. There were only 4 instances in which the application provided incorrect recommendations compared with the established protocols. System Usability Scale surveys of the CHWs suggested marginally acceptable usability (mean score of 62.1).^44^ Subgroup analysis suggested that CHWs who had at least some high school level education, who used smartphones regularly, and who had more experience with the application found the application easier to use. While we elicited feedback from CHWs at all points of application development and deployment, this feedback was dominated by the coordinators of the CHW program, who were generally better educated and had more experience in conducting diabetes visits. Thus, increasing “rank and file” CHW involvement in application development is one potential strategy to improve usability. CHWs also noted the tendency of the application to lag, negatively impacting usability. Our use of low-end Android devices likely accounts for this because we have found the application to work much faster on higher-performance devices. Fortunately, continued progress in smartphone development has meant that budget devices manufactured today are equivalent to flagship devices 2–3 years ago.^50^

The application provided reliable recommendations, with CHWs and the reviewing physician agreeing with the application-recommended medication dosing greater than 90% of the time.

WHO and other global health policy leaders have recognized the potential of mobile CDS tools to mitigate a lack of highly trained health care workers and supportive infrastructure and to improve the quality of care through the use of algorithmic protocols.^38^ ^,^ ^51^ These organizations have called for more rigorous evaluation of such mHealth interventions.^51^ ^,^ ^52^ Our experience in rural Guatemala adds to the evidence base supporting the use of mobile CDS to assist CHWs with chronic disease management and could be adapted for diabetes management in similar LMIC settings. This approach could also be applied to other chronic diseases amenable to algorithmic care, such as hypertension. We will freely share the application to allow others to build upon our work.

Integration with the greater health system is integral to the success of mobile health applications.^51^ ^,^ ^53^ ^,^ ^54^ While our program does not directly interface with the government health system in Guatemala at this time, such regional or national partnerships would be essential for effective scale-up. TulaSalud, a nongovernmental organization working in the northern highlands of Guatemala, provides a model for effective scale-up in collaboration with the Ministry of Health and other health care organizations.^55^ Using the CommCare platform, they have developed and deployed mobile applications to assist CHWs in maternal and child health initiatives, and enable care coordination with the Ministry of Health, across a service area of 3.4 million people.

### Medication Titration, Attenuation of Diabetes Control, and Medication Side Effects

It is possible that simply establishing consistent medication therapy through free provision of medications and regular follow-up, regardless of dose titration, accounted for improvements in glycemic control. Other studies of diabetes management in LMICs have shown marked improvements in A1c resulting from reconstitution of medication therapy, particularly when baseline A1c is high.^56^ ^,^ ^57^ However, 2 factors support the importance of the titration algorithm in our program. First, most patients (82%) reported that they were taking medications at the time of enrollment. Thus, subsequent improvements in glycemic control suggest that medication optimization and not merely initiation played a role for most patients. Secondly, median doses of metformin and glyburide increased significantly during the follow-up period.

Our data suggest possible attenuation of program effects on glycemic control over time. Although the reduction in A1c remained significant at 12 months after enrollment, A1c reduction peaked at 6 months and trended back towards baseline after this point. So-called “secondary failure” of hypoglycemic medications—a reduction in efficacy over time, particularly for glyburide and in patients with prior long-term, high-dose treatment^58^—could also contribute to long-term attenuation of improvements in glycemic control.

Based on our data, the safety of the intervention was comparable to routine diabetes care delivered in other contexts. Patients experienced metformin side effects requiring dosage reduction at 3.9% of metformin-exposed visits. This outcome is comparable to clinical trials of metformin, which generally report a 5% prevalence of metformin intolerance.^59^ Thirty-six percent of glyburide-exposed patients experienced probable hypoglycemia symptoms or documented hypoglycemia, with a mean of 0.9 events per patient-year of therapy. None of these episodes were severe. Published estimates of the frequency of hypoglycemia attributable to glyburide and other sulfonylureas vary widely based on event definitions.^60^ ^–^ ^64^ A prospective study of 383 patients that used a definition of hypoglycemia similar to ours (patient report of hypoglycemia symptoms or documented glucose measurement in the hypoglycemic range) found a similar prevalence (39%) and incidence (1.92 events per person-year) of hypoglycemia in patients taking sulfonylureas.^65^

Based on our data, the safety of the intervention was comparable to routine diabetes care delivered in other contexts.

### Diabetes Self-Care Counseling

While patients enrolled for at least 6 months had higher SDSCA scores than newly enrolled patients in several self-care categories, these differences were only statistically significant for foot care and dedicated exercise (with exercise scores actually better in the newly enrolled group). Additionally, when we repeated this questionnaire with these newly enrolled patients after 6 months, there were no statistically significant improvements. Sample size was very small, including only 5 patients for repeated SDSCA questionnaires, so it is difficult to reach any conclusions on the effectiveness of CHW-delivered lifestyle counseling. However, the lack of significant change suggests that counseling may need to be intensified and optimized. Two other interventions in which CHWs and diabetes educators provided self-care counseling to indigenous Guatemalans with diabetes have reported significant improvements in glycemic control.^57^ ^,^ ^66^ Of note, both of these interventions were relatively intensive, with weekly visits in one intervention^66^ and mean counseling time of 10 hours over a 9-month period in the other.^57^ In contrast, visits in our program occur monthly and typically include approximately 10 minutes of diabetes self-care counseling.

### Program Costs

The estimated cost of this program is less than that reported for a nurse-led diabetes program in Guatemala: US$118 versus US$220 per patient, per year.^33^ However, this program provided more comprehensive services, including insulin and hypertension treatment. The cost of our program is also comparable to data from a recent systematic review of the cost of diabetes treatment in LMICs, which reported average annual treatment costs ranging from US$29.91 to US$237.38 per person.^67^

### Limitations

The primary limitation of this study is the lack of a control group. A future study comparing CHW-led care with physician, midlevel provider, or nurse-led care is necessary to determine the efficacy of our approach versus standard practice. Another limitation of our analysis was the substitution of inferred values for A1c and glucose when measurements fell outside the range of the measurement devices. This injects a degree of uncertainty into the calculated changes in mean A1c and glucose throughout the study. However, sensitivity analysis showed that changes in A1c were robust to this limitation in measurement. In addition, improvements in the proportion of patients meeting A1c goals were not affected by this measurement uncertainty, and this outcome supports the efficacy of the program in improving glycemic control.

Another issue inherent in A1c measurement is the effect of anemia, hemoglobinopathies, and other metabolic abnormalities.^68^ While hemoglobinopathies are rare in indigenous populations of the Americas,^69^ anemia (primarily iron-deficiency anemia) affects more than 20% of women of childbearing age in Guatemala.^70^ We did not screen subjects for anemia in this study, so we are unable to assess the potential effect of anemia on our results. However, the primary outcomes in this study were longitudinal with each subject acting as their own control, mitigating the potential effect of skewed A1c results due to anemia in our analysis.

Our study population was mostly women (82%). The “men’s health gap”—reduced health care utilization and poorer health outcomes among men compared to women—is an important global phenomenon.^71^ Other diabetes interventions in rural Guatemala have also struggled to recruit and retain men.^33^ ^,^ ^66^ The low participation levels of men are likely multifactorial,^71^ but in our experience the predominantly agricultural nature of men’s work in these communities, entailing long hours and lengthy travel to the fields, is a key factor. Despite offering home visits on weekends, we were unable to overcome these barriers. Further research is needed on how to improve outreach to men in rural Guatemala and similar contexts.

Due to a low referral completion rate, relatively few referrals for certain complications of diabetes (such as chest pain and vision problems), and lack of advanced diagnostic testing capabilities at the referral hospital, it is difficult to assess the accuracy and efficacy of our protocols for detection, management, and referral of potential diabetes complications. Although we did not have renal function testing available for our entire patient population to validate our algorithm for identifying patients at higher risk of renal impairment, 25.7% of patients who completed renal function testing had at least some degree of renal function impairment (GFR <60 mL/min/1.73 m^2^) and 11.4% had significant renal impairment (GFR <30 mL/min/1.73 m^2^). This is similar to the prevalence of decreased GFR in type 2 diabetics (22%) estimated from a large global study completed in 2006.^72^ Thus, even though we have testing data available for renal function, it is difficult to assess the effectiveness of our algorithm in identifying high-risk patients.

Finally, we designed this program and the CDS application to fit our specific context of rural Guatemala and the specific resources and capacity of our local partner, which may make our findings less generalizable to other settings. While we are hopeful that others will be able to learn from our experience and to use the application, significant modifications may be required for our model to be used elsewhere.

## CONCLUSIONS

A novel CHW-led diabetes program enabled by mobile CDS technology led to improvements in diabetes control for a rural Guatemalan population. A task-sharing model using nonphysician health care workers assisted by mHealth applications holds promise for improving the care of diabetes and other noncommunicable diseases in LMICs, which represent a crucial health challenge of the 21st century. Further work is needed to determine the efficacy of this approach compared with standard care, to enhance the application to allow for the delivery of more comprehensive diabetes management, and to better support lifestyle changes through enhanced counseling and interventions to improve the nutritional environment.

## Supplementary Material

20-00076-Duffy-Supplement.pdf

## Notes

**En español**

**Usando Trabajadores Comunitarios de la Salud y una Aplicación de Smartphone para Mejorar el Control de la Diabetes en una Zona Rural de Guatemala**

**Hallazgos claves**

- Una aplicación para smartphones que brinda apoyo algorítmico a las decisiones clínicas permitió a los trabajadores comunitarios de salud mejorar el control de la diabetes en un grupo de pacientes en una zona rural de Guatemala.

**Implicaciones claves**

- Los administradores de programas deben considerar equipar a los trabajadores comunitarios de salud con aplicaciones de apoyo a la toma de decisiones clínicas para permitir el compartir de tareas para el manejo de enfermedades crónicas.
- Los investigadores deben examinar la eficacia de esta estrategia para enfermedades crónicas distintas de la diabetes y compararlo con los modelos tradicionales de cuidado médico.

**Resumen**

**Antecedentes:** La prevalencia mundial de diabetes casi se ha duplicado desde 1980. Setenta y cinco por ciento de los pacientes con diabetes viven en países de ingresos bajos y medianos, como Guatemala, donde los sistemas de atención médica a menudo están mal equipados para el manejo de enfermedades crónicas. Los trabajadores comunitarios de la salud (TCS) y la tecnología de salud móvil se han aplicado cada vez más a la epidemia de diabetes en estos entornos, aunque principalmente en funciones de apoyo más que en el manejo directo de la diabetes. Buscamos mejorar la atención de la diabetes en las zonas rurales de Guatemala mediante el desarrollo de un programa de diabetes dirigido por los TCS y una aplicación para smartphones para brindarles apoyo en la toma de decisiones clínicas.

**Métodos:** Trabajamos con nuestros socios locales para desarrollar un modelo de programa y la aplicación para smartphones (utilizando la plataforma CommCare) y para capacitar a los TCS. Reclutamos pacientes con diabetes tipo 2 que vivían en comunidades rurales. La evaluación del programa utilizó un diseño pre-post de un solo grupo. Los resultados primarios fueron la hemoglobina A1c y el porcentaje de pacientes que alcanzaron los objetivos de A1c en comparación con el valor inicial. También seguimos una variedad de métricas de procesos, incluyendo la confiabilidad de la aplicación.

**Resultados:** Ochenta y nueve pacientes fueron inscritos durante el período de estudio. El porcentaje de hemoglobina A1c disminuyó significativamente a los 3 meses (−1,0; IC del 95%: −1,7 a −0,6), 6 meses (−1,5; IC del 95%: −2,2 a −0,8), 9 meses (−1,3; IC del 95%: −2,0 a −0,6) y 12 meses (−1,0; IC del 95%: −1,7 a −0,4). El porcentaje de pacientes con A1c ≤ 8% aumentó significativamente a los 3 meses (23,6% a 44,4%, *P*= 0,007), 6 meses (22,0% a 44,0%, *P*= 0,015) y 9 meses (23,9% a 45,7%, *P*= 0,03). Los TCS y los médicos supervisores estuvieron de acuerdo con las recomendaciones de la aplicación para el uso de medicamentos más que el 90% del tiempo.

**Conclusión**: Nuestros resultados sugieren que los TCS pueden manejar la diabetes de forma segura y eficaz con la ayuda de una aplicación para smartphones y la supervisión médica remota. Este modelo debe evaluarse frente a otros estándares de cuidado médico y podría adaptarse a otros entornos de escasos recursos otras enfermedades crónicas.

## Notes

**Peer Reviewed**

**First published online:** December 4, 2020

**Funding:** The University of Wisconsin Global Health Institute provided seed grant funding for this project. Dr. Duffy was also supported by the National Research Service Award Primary Care Postdoctoral Research Traineeship awarded by the National Institutes of Health, Health Resources Services Administration (HRSA grant T32 HP10010) during a portion of the time spent working on this project.
