# Supplementary material for: Using Community Health Workers and a Smartphone Application to Improve Diabetes Control in Rural Guatemala
Source: Glob Health Sci Pract. 2020 Dec 23;8(4):699–720. doi: 10.9745/GHSP-D-20-00076 (PMC7784066; doi:10.9745/GHSP-D-20-00076)
Supplement: 20-00076-Duffy-Supplement.pdf [file 20-00076-Duffy-Supplement.pdf]

Supplement to: Duffy S, Norton D, Kelly M, et al. Using community health workers and a smartphone application to improve diabetes control in rural Guatemala. *Glob Health Sci Pract*. 2020;8(4). https://doi.org/10.9745/GHSP-D-20-00076

Supplemental Table 1 – GAMM results (linear elements)

Outcome	Covariate	Estimate	Std. Error	t value	p value
A1C (%)	Male	-0.362	0.478	-0.758	0.449
	Age (years)	***-0.0456***	***0.0146***	***-3.12***	***0.00199***
	diabetes diagnosis	***0.0731***	***0.0315***	***2.32***	***0.0209***
Glucose (natural-log ml/dl)	Male	-0.132	0.0829	-1.59	0.112
	Age (years)	***-0.00756***	***0.00251***	***-3.01***	***0.00271***
	diabetes diagnosis	***0.0181***	***0.00544***	***3.33***	***0.000919***
	Fasting	***-0.416***	***0.0261***	***-16***	***1.03E-50***
Systolic BP (mmHg)	Male	-1.7	3.9	-0.437	0.663
	Age (years)	***0.569***	***0.116***	***4.9***	***1.12E-06***
	Years since diabetes diagnosis	0.2	0.257	0.777	0.437
Diastolic BP (mmHg)	Male	0.996	1.8	0.553	0.58
	Age (years)	0.00393	0.0537	0.0733	0.942
	Years since diabetes diagnosis	0.0038	0.119	0.0318	0.975
Weight (lbs)	Male	8.63	6.3	1.37	0.171
	Age (years)	-0.264	0.183	-1.44	0.15
	Years since diabetes diagnosis	0.11	0.418	0.262	0.793
Waist-circumference (cm)	Male	-0.423	2.14	-0.197	0.844
	Age (years)	0.105	0.0633	1.66	0.0973
	Years since diabetes diagnosis	-0.114	0.142	-0.805	0.421
BMI (kg/m^2)	Male	-2.14	1.28	-1.67	0.0957
	Age (years)	0.0161	0.0373	0.432	0.666
	Years since diabetes diagnosis	-0.0299	0.085	-0.353	0.725
**Outcome**	**Covariate**	**OR estimate**	**OR 95% LCL**	**OR 95% UCL**	**p value**
A1C control (A1C <= 8%)	Male	1.68	0.63	4.46	0.301
	Age (years)	***1.05***	***1.01***	***1.08***	***0.00454***
	diabetes diagnosis	***0.896***	***0.829***	***0.968***	***0.00526***
A1C goal (A1C <= subject's goal)	Male	3.11	0.94	10.3	0.063
	Age (years)	***1.08***	***1.03***	***1.12***	***0.000475***
	diabetes diagnosis	***0.889***	***0.802***	***0.986***	***0.0253***

Supplement to: Duffy S, Norton D, Kelly M, et al. Using community health workers and a smartphone application to improve diabetes control in rural Guatemala. *Glob Health Sci Pract*. 2020;8(4). https://doi.org/10.9745/GHSP-D-20-00076

Supplemental Table 2 – GAMM results (non-linear elements)

Outcome	EDF	F	p-value
A1C (%)	***4.09***	***8.1***	***2.80E-06***
Glucose (natural-log ml/dl)	***4.58***	***4.23***	***0.00206***
Systolic BP (mmHg)	1	2.83	0.0926
Diastolic BP (mmHg)	2.02	1.08	0.349
Weight (lbs)	***4.39***	***6.47***	***2.54E-05***
Waist-circumference (cm)	2.07	1.77	0.151
BMI (kg/m^2)	***3.63***	***5.8***	***0.000224***
**Outcome**	**EDF**	**Chi-square**	**p-value**
A1C control (A1C <= 8%)	2.63	10.1	0.0676
A1C goal (A1C <= subject's goal)	2.28	6.07	0.118

EDF: effective degrees of freedom of the estimated non-linear function
